# Quantification of injury burden using multiple data sources: a longitudinal study

**DOI:** 10.1038/s41598-021-82799-9

**Published:** 2021-02-04

**Authors:** Keith T. S. Tung, Frederick K. Ho, Wilfred H. S. Wong, Rosa S. Wong, Matthew S. H. Tsui, Paul Ho, Chak Wah Kam, Esther W. Y. Chan, Gilberto K. K. Leung, Ko Ling Chan, Chun Bong Chow, Patrick Ip

**Affiliations:** 1 Department of Paediatrics and Adolescent Medicine, Queen Mary Hospital, University of Hong Kong, Room 123, New Clinical Building, Pok Fu Lam, Hong Kong, China; 2 Institute of Health and Wellbeing, University of Glasgow, Glasgow, UK; 3 Department of Accident and Emergency, Queen Mary Hospital, Hong Kong, China; 4 Department of Accident and Emergency, Queen Elizabeth Hospital, Hong Kong, China; 5 Department of Accident and Emergency, Tuen Mun Hospital, Hong Kong, China; 6 Centre for Safe Medication Practice and Research, University of Hong Kong, Hong Kong, China; 7 Department of Surgery, University of Hong Kong, Hong Kong, China; 8 Department of Applied Social Sciences, Hong Kong Polytechnic University, Hong Kong, China

**Keywords:** Quality of life, Trauma, Epidemiology, Outcomes research

## Abstract

Quantification of injury burden is vital for injury prevention, as it provides a guide for setting policies and priorities. This study generated a set of Hong Kong specific disability weights (DWs) derived from patient experiences and hospital records. Patients were recruited from the Accident and Emergency Department (AED) of three major trauma centers in Hong Kong between September 2014 and December 2015 and subsequently interviewed with a focus on health-related quality of life at most three times over a 12-month period. These patient-reported data were then used for estimation of DWs. The burden of injury was determined using the mortality and inpatient data from 2001 to 2012 and then compared with those reported in the UK Burden of Injury (UKBOI) and global burden of diseases (GBD) studies. There were 22,856 mortality cases and 817,953 morbidity cases caused by injuries, in total contributing to 1,027,641 disability-adjusted life years (DALYs) in the 12-year study timeframe. Estimates for DALYs per 100,000 in Hong Kong amounted to 1192, compared with 2924 in UKBOI and 3459 in GBD. Our findings support the use of multiple data sources including patient-reported data and hospital records for estimation of injury burden.

## Introduction

Intentional and unintentional injuries are the leading causes of global morbidity, mortality and premature death^1^, causing around 5 million deaths annually (9% of the global mortality). Injury is also a significant health problem in Hong Kong. Each year around 6.2% of the population reported functional limitations caused by injuries^2^. Quantification of injury burden is important for surveillance and prevention activities. However, few studies have estimated the burden of injury at population level. This estimation process is complex and influenced by high heterogeneity across injury types and situations such as injury severity, recovery duration and outcomes. The injury pyramid by Wadman et al.^3^ illustrated the interrelationship between fatal and non-fatal injuries which should be examined together, as their effects can be addictive and result in various health problems ranging from temporary pain and inconvenience to lifelong disabilities.

The 1990 Global Burden of Diseases, Injuries and Risk Factors (GBD) Study was the first to develop and utilize a comprehensive assessment method to estimate population-level burden caused by injuries^4^. The central component of the GBD methodology is the calculation of disability-adjusted life years (DALYs) from expert opinions and clinical data. The DALYs, which has been an important measure of healthcare burden since its first conceptualization in 1990s^4^, reflect the aggregated effects of both fatal and non-fatal injuries^5,6^. Specifically, these estimates are derived from overall health loss in a population which is equivalent to the sum of years lost due to premature mortality (YLL) and years lived with disability (YLD). The YLL represents the incidence of fatal injuries, whereas the YLD represents the healthy time lost due to injuries after considering the incidence of non-fatal injuries, disability weights (DWs) and duration of recovery or until death.

However, there has been debate over the best approach to estimation of DWs. Polinder et al.^7^ suggested in their review that many existing methods were insufficient to compute accurate DWs due to issues such as variations in injury assessment instruments, definition of incident and fatal cases, and lack of information in valuation of disability. For example, the GBD 1990 study derived DWs and duration of recovery for different injury groups from panel studies and expert opinions rather than direct measurement. The GBD 1990 study, although considered a major milestone in quantification of healthcare burden, had limited data on injury incidences and DWs which may potentially underestimate burden of injury^8,9^. To address these limitations, it has been suggested that valid estimation of DWs requires a comprehensive set of epidemiological data including injury types, severity, duration, and outcomes from multiple sources^10,11^.

The UK burden of injuries (UKBOI) study, on the other hand, employed a mixed methods design, in which data from hospital and injured individuals recruited from multiple centers were incorporated to quantify population-level injury burden^6,8,9^. Results of the UKBOI study showed that, compared to hospital records alone, the use of patent-reported data can give more accurate DW estimates. In view of these results, the data sources for derivation of DWs have been changed from judgement of healthcare professionals in GBD 1990 to use of general public judgment of symptom severity in GBD 2010 and patient-reported data in GBD 2013^12–14^. Recent studies also used patient-reported outcomes for derivation of DWs^15–18^. However, most studies were conducted in Caucasian populations, and only one study was conducted in Asian population^15^. The lack of culturally relevant DWs can hinder the development of effective injury prevention strategies for these understudied populations, particularly when patients’ perception of the impact of an injury can vary between cultural settings^19,20^. For example, Chinese patients tend to prefer collectivism over individualism, and such preference may differentiate Chinese and western patient perceptions and reports of pain and other symptom severity following the same injury incidence^20^. Therefore, this study adopted multiple estimation methods to generate a set of Hong Kong specific DWs derived from patient-reported data and hospital records. This study also assessed differences in injury burden between western and Chinese cultures by comparing estimates of injury burden with Hong Kong specific DWs and those reported in the GBD and UKBOI studies. The main outcomes of the current study are therefore the Hong Kong specific DWs and DALYs estimated from these DWs.

## Results

### Participants

A total of 1924 patients were recruited from the Queen Elizabeth Hospital (n = 218, 11.3%), Queen Mary Hospital (n = 1,372, 71.3%), and Tuen Mun Hospital (n = 334, 17.4%). The average age of the recruited patients was 50.7 years, and 50.0% of them were males. 673 of them were (35.0%) admitted to the hospital after AED attendance. Workplace was the most frequent location of injury (24.7%), followed by home (22.6%). The most common mechanism causing injuries was fall (49.4%) followed by being hit/struck (14.0%). Most of the injury types included in this study were able to be mapped to the EUROCOST injury types. Table 1 shows the distribution of injury types by age group among the recruited patients.

**Table 1:** Distribution of study participants among EUROCOST injury types, and estimation of disability weights and proportion of lifelong consequences.

EUROCOST injury types	12–24	25–59	60+	Total	DWs (acute)	DWs (lifelong)	Proportion of lifelong consequence (%)
Concussion	9	28	36	73	0.044	0.005	6.7
Other skull-brain injury	3	10	10	23	0.079	0.001	6.3
Open wound head	5	16	24	45	0.010	0.000	0.0
Eye injury	4	22	7	33	0.025	0.004	2.5
Fracture facial bones	5	5	4	14	0.151	0.026	21.1
Open wound face	7	19	17	43	0.043	0.000	8.4
Fracture/dislocation/strain/sprain vertebrae/spine	8	53	23	84	0.056	0.002	11.0
Whiplash, neck sprain, distortion cervical spine	3	15	3	21	0.136	0.016	32.0
Spinal cord injury	0	1	0	1	0.654	0.394	100.0
Internal organ injury	0	0	0	0	0.216	0.055	59.1
Fracture rib/sternum	0	15	12	27	0.092	0.000	6.1
Fracture clavicle/scapula	0	8	8	16	0.141	0.021	12.6
Fracture upper arm	0	5	20	25	0.183	0.002	25.1
Fracture elbow/forearm	5	15	14	34	0.174	0.016	12.5
Fracture wrist	4	45	50	99	0.138	0.002	10.5
Fracture hand/fingers	2	21	10	33	0.115	0.032	22.4
Dislocation/sprain/strain shoulder/elbow	7	20	8	35	0.075	0.014	13.6
Dislocation/sprain/strain wrist/hand/fingers	8	28	10	46	0.083	0.013	14.5
Injury of upper extremity nerves	0	0	0	0	0.142	0.022	34.6
Complex soft tissue injury upper extremity	0	8	0	8	0.123	0.003	17.5
Fracture pelvis	1	3	3	7	0.041	0.001	8.9
Fracture hip	0	4	53	57	0.150	0.077	57.4
Fracture femur shaft	0	3	4	7	0.286	0.080	72.8
Fracture knee/lower leg	3	30	31	64	0.222	0.029	31.6
Fracture ankle	7	42	5	54	0.168	0.028	18.1
Fracture foot/toes	5	32	19	56	0.141	0.020	12.7
Dislocation/sprain/strain knee	11	42	27	80	0.069	0.006	13.1
Dislocation/sprain/strain ankle/foot	34	64	18	116	0.065	0.000	10.0
Dislocation/sprain/strain hip	2	6	4	12	0.060	0.018	21.1
Injury of lower extremity nerves	0	0	0	0	0.230	0.060	100.0
Complex soft tissue injury lower extremities	2	5	0	7	0.198	0.001	0.0
Superficial injury, incl. contusions	47	199	120	366	0.037	0.005	8.3
Open wounds	34	101	30	165	0.030	0.002	5.2
Burns	6	19	5	30	0.030	0.016	4.9
Poisoning	5	15	4	24	0.025	0.000	21.1
Foreign body	5	15	6	26	0.000	0.000	0.0
Other injury	10	39	16	65	0.047	0.008	11.9
Total	244	959	614	1817			

Remarks: 85 of them cannot be categorized into the 39 EUROCOST injury group; 22 patients did not provide sufficient data to compute disability weights.

*DW* disability weight.

At one month after the injury, 1728 patients (89.8%) were contacted via phone calls. Around 45% (775) of them still reported functional limitations due to injury. Unrecovered patients were further contacted at 4-month post injury, of whom 337 (43.5%) were still affected by the injury. All patients were contacted again at 12 months post-injury with a response rate of 80.0%. Around 13.1% (201) of them were still unrecovered and were considered as having a lifelong consequence due to injury.

### Disability weights (DWs)

**Figure 1:**
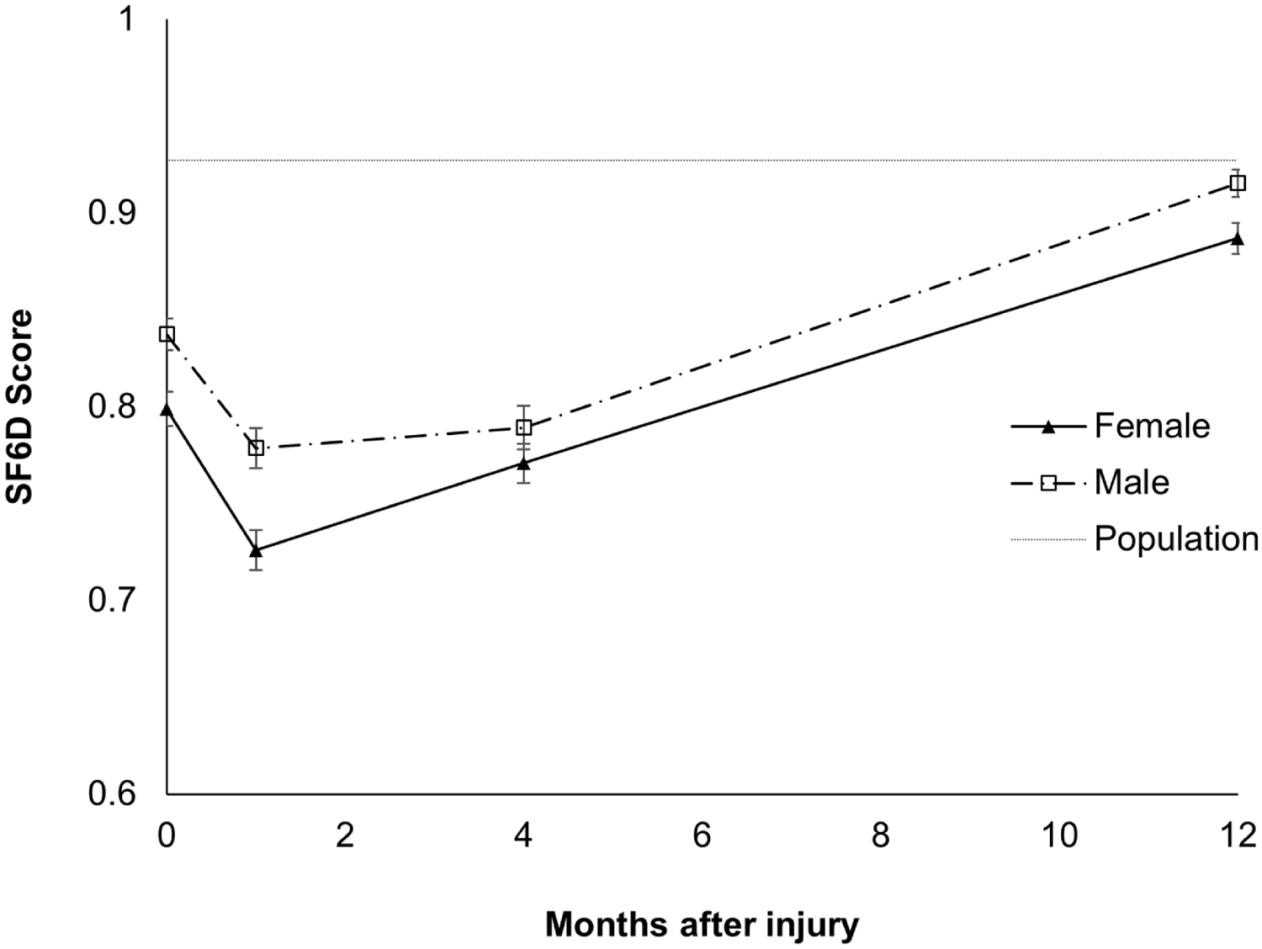
SF6D single utility score among recruited patients at 1-, 4-, 12-month post injury.

Figure 1 displays the overall changes of the utility index during the study period. It was found that the index scored low at 1-month post-injury and increased steadily at 4 and 12 months, with male patients reporting higher scores at all time points.

Length of hospital stay was used as a proxy for estimation of DWs of rare injury types. Linear regression analyses were conducted to examine the association between length of stay and DWs, which showed that DWs at all time points can be predicted by length of stay with regression coefficients of 0.004 (1st and 4th months) and 0.002 (12th month) (all p < 0.001). When the measures were standardized to their SDs, the coefficients were 0.24 and 0.30, respectively, indicating small-to-medium effect sizes. Based on these results, DWs were estimated by the rate of 0.004 at 1st and 4th months and 0.002 at 12th month for hospitalization due to rare injury types. Acute and lifelong DWs together with the proportion of lifelong consequences of each EUROCOST injury type were computed as shown in Table 1. The injury type with the highest DW was spinal cord injury (0.654), followed by fracture of femur shaft (0.286) and injury of lower extremity nerves (0.230). Moreover, the lifelong DWs were generally lower than the acute DWs for most of the injury cases.

### YLLs, YLDs and DALYs

In total there were 22,856 cases of injury mortality and 817,953 cases of injury morbidity during the period of 2001–2012. The total number and rate of injury episodes are shown in Supplementary Table 1. The most common injury type was skull-brain injury other than concussion, followed by hip fracture. Table 2 shows the population-level YLLs and YLDs by sex, age group, and year using the DWs derived from our study. During the 12-year timeframe of this study, injury caused a total of 1,021,815 DALYs in Hong Kong, which is equivalent to 85,151 DALYs on a yearly basis. Injury-related burden of disease was 1192 DALYs per 100,000 population. As shown in Table 2, males contributed more to DALYs (624,844) than females (396,971), but the trend was decreasing from 2001 to 2012. The average YLL to YLD ratio in this study was 2.3, which was higher than the estimate of the UKBOI study (0.2) yet lower than that of the GBD study (5.7). Furthermore, the DALYs reported in this study (1192 per 100,000) were much lower than that in the UKBOI (2924 per 100,000) and the GBD Study (3459 per 100,000) (Table 3).

**Table 2:** DALYs, YLL, YLD (acute and lifelong) and YLL:YLD ratio according to sex, age and year stratification.

	DALYs	YLL	YLD (acute)	YLD (lifelong)	YLL:YLD ratio
**Sex**					
Male	624,844	459,626	64,663	100,555	2.78
Female	396,971	253,390	64,009	79,573	1.76
**Age**					
0–14	40,957	23,967	6994	9995	1.41
15–59	734,971	579,912	54,945	100,114	3.74
60 or above	245,887	109,136	66,732	70,019	0.80
**Year**					
2001	91,562	65,080	10,745	15,737	2.46
2002	93,451	67,891	10,452	15,108	2.66
2003	89,874	66,469	9440	13,966	2.84
2004	95,215	71,127	9771	14,316	2.95
2005	91,617	68,438	9495	13,684	2.95
2006	83,991	60,714	9524	13,753	2.61
2007	78,471	55,874	9560	13,037	2.47
2008	77,016	53,347	10,186	13,483	2.25
2009	84,216	57,504	11,309	15,403	2.15
2010	85,802	56,069	12,441	17,292	1.89
2011	74,592	44,772	12,712	17,108	1.50
2012	76,008	45,731	13,037	17,240	1.51

*YLDs* year lived with disability; *YLLs* years of life lost, *DALYs* disability-adjusted life years.

**Table 3:** YLDs, YLLs, YLL:YLD ratio and DALYs comparisons with overseas studies (per 100,000 population).

Country, Year	YLLs per 100,000	YLDs per 100,000	YLL:YLD ratio	DALYs per 100,000
United Kingdom, 2005^8^	526	2398	0.2	2924
Global, 2013^35^	2945	515	5.7	3459
Hong Kong, 2001–2012	867	286	2.3	1192

*YLDs* year lived with disability, *YLLs* years of life lost, *DALYs* disability-adjusted life years.

## Discussion

This study utilized and incorporated multiple sources of data from patients and hospitals to quantify the burden of injury at population level. A local prospective cohort of about 2000 injured patients was established to generate a set of Hong Kong specific DWs to allow for estimation of injury burden indicated by DALYs in Hong Kong. We found that the DALYs reported in this study was lower than those reported in the UKBOI and GBD studies. Moreover, mortality was found to be the major contributor to injury burden in Hong Kong as indicated by a high YLL to YLD ratio, and most injuries imposed relatively short-term burden on patients^21^.

Although assessment of injury burden is important for policy formulation and setting priorities, there has been no consensus on the method that is both valid and reliable for estimation of DWs attributable to injury in the scientific literature. While the approach used in the GBD 1990 study^1^ heavily relied on the opinions from an expert panel, we used empirical data collected from local injured patients to develop a set of DWs that were culturally sensitive and relevant to the Hong Kong setting. This approach was similar to that used in the UKBOI study^6,8^ which utilised patient experiences-derived DWs and healthcare data to estimate the DALYs attributable to injury. The use of multiple data sources to quantify health losses attributable to fatal and non-fatal injuries has been suggested to provide the most accurate estimates of injury burden^8^. The establishment of Hong Kong-specific DWs in this study can help local policy makers in making accurate decisions and predictions on injury burden and trends in Hong Kong. The findings also inform future prevention strategies and priorities setting by highlighting the most disabling injury types and the patient groups with unmet needs.

However, estimation of patient experiences-derived DWs requires the collection of pre-injury HRQoL data. This study had considered three methods to evaluate pre-injury HRQOL, including patient recall of pre-injury HRQoL level (Method 1), use of normative values from previous population-based studies (Method 2), and proxy evaluation of pre-injury HRQoL by averaging the utility indexes of recovered patients within the 12-month study period (Method 3). Compared to Method 1 and Method 2, Method 3 was considered more reliable in terms of reflecting the true value of injury-related HRQoL loss. It is because Method 1 may involve recall bias, as traumatic events could have altered patients’ perception of their own health states. Method 2 may also generate biased estimates because of the potential socio-demographic differences between injured patients and the general population, as it has been reported that injuries occur more frequently in individuals from underprivileged social class^22^. As such, if using Method 2, patients suffering injuries may have experienced lower levels of pre-injury HRQoL than the general population which could potentially inflate the estimation of DWs. Method 3 computes DWs using the population mean utility index reported by recovered patients which should produce the most valid estimates, and thus we adopted this method to compute the Hong Kong specific DWs.

Most of the DWs in this study were found to be lower than those reported in other UK studies^1,6,8^, which could be explained by two possibilities. One possibility is that injuries in Hong Kong might be less severe than those in the UK. The other possibility pertains to cultural differences between Hong Kong and the UK, as the DWs in this study were estimated from patient-reported experiences following injury incidents which could be subject to cultural influences^19,20^. For example, due to differences in preference for individualism and collectivism between the western and Chinese cultures^20^, a patient with broken arm in western societies may regard themselves to have higher physical limitations when compared to their counterparts in Chinese societies. Notably, for most injury types, we cannot make direct comparison of DWs between the UKBOI study and the current study, as the UKBOI study used a 13-category injury classification system and yet we used the EUROCOST 39-category classification. For the remaining comparable subgroups such as upper and lower extremity fractures, the acute DWs for upper and lower extremity fractures in this study ranged from 0.115 to 0.183 and from 0.141 to 0.286 respectively, but the DWs of admitted cases due to upper and lower extremity fractures in the UKBOI study were 0.120 and 0.240^8^. We further compared the DWs in this study against findings from previous meta-analysis of six injury cohort studies on DWs derived from patient-reported data in five developed countries^16^. The median acute and lifelong DWs (interquartile ranges [IQRs]) reported in this meta-analysis^16^ were 0.127 (0.076–0.188) and 0.107 (0.04–0.171) respectively, when compared to the same estimates in our study of 0.092 (0.044–0.151) and 0.008 (0.002–0.022) respectively. Furthermore, 24 out of the 30 DWs generated in our study are lower than those reported in the meta-analysis^16^, majority of which are lifelong DWs. This could be due to the prompt response of emergency services and high quality of care in secondary and tertiary healthcare institutes in Hong Kong, which have been shown to have great influences on patient trauma outcomes^23^.

We found that compared to the UK, Hong Kong experienced greater injury burden due to mortality than to disability as indicated by a higher YLL:YLD ratio in this study than that reported in the UKBOI study. It has been reported that the estimation of the YLL:YLD ratio can be affected by a wide range of methodological factors, including DWs estimation, injury grouping, and data source^24^. This finding suggests that the nature of injury burden may vary across societies. In particular, Hong Kong has a very low injury mortality rate when compared to other populations^25,26^. The ratio therefore further suggests that the proportion of lifelong disability due to injury among Hong Kong residents could be even lower than that in other populations, possibly because the health care and medication administration systems in Hong Kong are of high quality, and most injuries are mild which do not necessarily result in hospital admission and thus contribute to smaller burden on local injured patients.

In addition, our study contributes to the literature by illustrating how to make use of multiple data sources to generate estimates of injury burden in Hong Kong. It should be noted that the DALYs reported in this study are indicative of injury burden for both mortality and inpatient cases. For instance, if a patient suffering from knee or lower leg fractures has an acute DW of 0.222 and lifelong DW of 0.029 at their first follow-up, these generated DWs can be combined with hospitalization data and AED records to generate YLD estimates attributable to knee or lower leg fractures. We can further quantify the level of burden due to knee or lower leg fractures by computing the DALYs using the associated YLD and the YLL estimates derived from mortality data. It is therefore important to adopt standardized injury surveillance measures within the healthcare system, particularly the inclusion of AED data, for routine collection and analysis of injury-related data. The injury surveillance model based on AED data of a hospital^27^ could be extended by adding a linkage with inpatient records^28^ which can provide more information about long-term injury outcomes and thus achieve higher accuracy in estimation of injury burden.

Findings of this study should be interpreted with the following caveats. First, our study may not be able to assess the burden of all types of injury. We attempted to overcome this issue by adopting the length of hospital stay as a proxy to estimate the DWs for rare injury conditions. The second limitation of this study is the restricted follow-up period up to 12 months. This might affect the accuracy of the lifelong DWs estimation, as our current calculation was carried out based on the assumption that patients who had not been recovered from the injury by 12 months would have lifelong consequences. Other studies also acknowledged the weaknesses of this approach^7,8,29,30^. In addition, the current method of DW estimation could be overestimated, as the loss in QoL might not be attributable to the injury episode. On the other hand, as the diagnosis data retrieved from the AED system was incomplete, we only included admission data in the calculation of DALYs, and this may potentially cause underestimation. However, the risk of underestimation should be low, as most patients with severe injuries should have been admitted to hospitals for treatment and observation. Finally, the lack of objective assessment of disability may also limit generalizability of the findings.

This study utilized and integrated patient-reported data, hospital data and mortality records to estimate the burden of injury at the population level. We found a unique set of DWs and DALYs that are specific to Hong Kong population and are different from those reported in the GBD and UKBOI studies. The results suggest that compared to expert panel-based estimation, patient-reported data might provide more reliable and valid estimates of the burden of injury which have implications for future injury prevention efforts. Moreover, the Hong Kong specific DWs generated in this study will be useful for surveillance and monitoring of local injury burden with higher accuracy and thus inform service planning and delivery approaches.

## Methods

### Study participants

This study recruited individuals with any medical histories of intentional or unintentional injuries from three major trauma centers in Hong Kong (Queen Elizabeth Hospital, Queen Mary Hospital and Tuen Mun Hospital) during the period of September 2014 to December 2015. These three hospitals were selected, as they are designated as trauma centers for three major areas in Hong Kong, namely the Hong Kong Island, Kowloon, and New Territories. The caseloads of these trauma centers are believed to be representative of the overall injury situation in Hong Kong. Quota sampling was adopted for recruitment to ensure the inclusion of different types of injury at various levels of severity. Individuals who attended the Accident and Emergency Department (AED) or admitted to the hospital due to injury during the study period were approached by trained research staff with the help of trauma nurses at the AED. After obtaining informed consent, patients were instructed to complete questionnaires on their demographics, details of the occurrence of injury, and their health-related quality of life (HRQoL) prior to the incidence of injury and were followed up over 12 months. They were subsequently interviewed by phone on recovery progress and HRQoL at 1- and 4-month post-injury or until full recovery was reported, whichever earlier. At 12-month post injury, telephone survey on health status was administered to all recruited patients, regardless of their recovery progress. Patients who did not respond to initial follow-up phone calls were contacted repeatedly via phone calls at different times of the day to minimize the study attrition rate. Patients’ hospitalization data and AED records in the follow-up period were extracted from the electronic health record sharing system under the local public hospital network.

Individuals aged below 12 years were excluded from this study due to the lack of appropriate measurement tool and Hong Kong-based population norm for evaluation of their HRQoL. Individuals who did not provide consent or were not Hong Kong permanent residents were also excluded.

### Measures and statistical analysis

The DALYs were used to estimate population burden of injury. DWs, YLLs and YLDs were calculated using self-reported patient experience data, hospital data, and mortality records.

#### Disability weights estimation

DW is essential for calculating the YLDs. It is the loss of quality of life following the injury and indicator of the severity of injury experienced by the patient. It is measured on a scale ranging from 0 (perfect health condition) to 1 (worst health condition, death)^31^. In this study, the DWs were derived from data on patients’ self-reported HRQoL which was measured by the 12-item Short-Form Health Survey (SF-12v2)^32^. The SF-12v2 is a widely used scale for assessing daily functions and can be used to compute a single utility index (SF6D) which is an indicator of health state^33^. In this study, patients could have at most three SF6Ds measured at 1-, 4-, and 12-month post injury. The follow-up would be terminated when they reported full recovery, and their subsequent SF6Ds would be assumed to remain the same. All the collected SF6D data from the recovered patients over the 12-month study period were averaged to generate the population mean index (PMI). The acute DWs were computed as the difference between the SF6D measured at 1-month post-injury and the PMI (${SF6D}_{1m}-PMI$), whereas the lifelong DWs were computed as the difference between the mean of all post-injury SF6D and the PMI $\left( {\frac{{SF6D_{1m} + SF6D_{4m} + SF6D_{12m} }}{3} - PMI} \right)$. The study was originally planned to recruit at least six patients for each age and injury category in order to ensure the 95% margin of error within 10% of mean. The plan was difficult to achieve particularly for certain combinations of age and injury type. We therefore used regression analysis to estimate the DWs using length of hospital stay based on the assumption that the length of stay was mainly driven by the level of disability.

#### Calculation of years-of-life-lost (YLLs) and years lived with disability (YLDs)

The mortality data, including date of death, sex, age at death, residential district and all causes of death, were obtained from the Hong Kong Department of Health. Gender- and sex- specific mortality rate due to injury from 2001 to 2012 according to the ICD-10 causes of death were obtained. The population-level YLLs due to injury were calculated by matching the mortality data with the gender- and sex-specific life expectancies extracted from the 2011 Life Table by the Census and Statistics Department. To quantify the YLD component of DALYs, hospitalization data and AED record related to injury were retrieved from Clinical Data Analysis and Report System (CDARS). The CDARS captures majority of the hospitalization and 24-h AED service data in Hong Kong, including length of hospital stay which is a recognized indicator of injury severity. The International Classification of Diseases, 9th revision (ICD-9) system for diagnosis^34^ was adopted in the CDARS to document the types of injuries. E-codes (diagnostic codes with first position as ‘E’) were also used to identify any external causes of AED admissions. The YLDs were computed as the product of injury incidence, DWs estimated from patient data, and duration of recovery or until death^24^. The short-term YLDs were computed as the product of DW_1-year_ and number of occurrences of the corresponding injury type, whereas the long-term YLDs were computed by multiplying the lifelong DWs with the prevalence of all injury types, the proportion of lifelong consequences after injury and the remaining life expectancy of the injured patients.

### Ethics approval

The study and consent procedures stated in the protocol of this study have been approved by the ethics committee of the Institutional Review Board of Hospital Authority in Hong Kong West Cluster (Ref. No.: UW 13-252), Kowloon Central Cluster (KC/KE-13-0148/ER-1), and New Territories West (1197/13). All methods of this study were performed in accordance with the relevant guidelines and regulations. Informed consent was obtained from all patients joining this study. For patients aged below 18 years, informed consent was obtained from both the patients themselves and their guardians.

## Supplementary Information

Supplementary Table 1.

## Data Availability

The data that support the findings of this study are available from Hong Kong Department of Health but restrictions apply to the availability of these data, which were used under license for the current study, and so are not publicly available. Data are however available from the authors upon reasonable request and with permission of Hong Kong Department of Health.
