# Supplementary material for: Quantification of injury burden using multiple data sources: a longitudinal study
Source: Sci Rep. 2021 Feb 4;11:3078. doi: 10.1038/s41598-021-82799-9 (PMC7862366; doi:10.1038/s41598-021-82799-9)
Supplement: Supplementary file 1 — Supplementary Table 1. [file 41598_2021_82799_MOESM1_ESM.docx]

**Quantification of injury burden using multiple data sources: A longitudinal study**

Keith T.S. Tung^1^, Frederick K. Ho^2,1^, Wilfred H.S.Wong^1^,

Rosa S. Wong^1^, Matthew Tsui^3^, Paul Ho^4^, Chak Wah Kam^5^,

Esther W.Y. Chan^6^, Gilberto K.K. Leung^7^, Ko Ling Chan^8^,

Chun Bong Chow^1^, Patrick Ip^1^

1. Department of Paediatrics and Adolescent Medicine, University of Hong Kong, Hong Kong
2. Institute of Health and Wellbeing, University of Glasgow, Glasgow, United Kingdom
3. Department of Accident and Emergency, Queen Mary Hospital, Hong Kong
4. Department of Accident and Emergency, Queen Elizabeth Hospital, Hong Kong
5. Department of Accident and Emergency, Tuen Mun Hospital, Hong Kong
6. Centre for Safe Medication Practice and Research, University of Hong Kong, Hong Kong
7. Department of Surgery, University of Hong Kong, Hong Kong
8. Department of Applied Social Sciences, Hong Kong Polytechnic University, Hong Kong

Corresponding author: Dr Patrick Ip

Room 123, New Clinical Building, Queen Mary Hospital, Pokfulam, Hong Kong

Email: [patricip@hku.hk](mailto:patricip@hku.hk)

Tel: +852 2255 4090

Fax: +852 2255 4089

**Supplementary Table 1**. Numbers and rates of injury episodes by EUROCOST injury types

|  | **Number** | **Rate per 100,000** |
| --- | --- | --- |
| Concussion | 12238 | 14.8 |
| Other skull-brain injury | 109010 | 131.6 |
| Open wound head | 5926 | 7.2 |
| Eye injury | 3961 | 4.8 |
| Fracture facial bones | 11160 | 13.5 |
| Open wound face | 11438 | 13.8 |
| Fracture/dislocation/strain/sprain vertebrae/spine | 17634 | 21.3 |
| Whiplash, neck sprain, distortion cervical spine | 5334 | 6.4 |
| Spinal cord injury | 3535 | 4.3 |
| Internal organ injury | 6124 | 7.4 |
| Fracture rib/sternum | 12913 | 15.6 |
| Fracture clavicle/scapula | 7529 | 9.1 |
| Fracture upper arm | 17976 | 21.7 |
| Fracture elbow/forearm | 30286 | 36.6 |
| Fracture wrist | 48585 | 58.6 |
| Fracture hand/fingers | 26209 | 31.6 |
| Dislocation/sprain/strain shoulder/elbow | 11387 | 13.7 |
| Dislocation/sprain/strain wrist/hand/fingers | 5128 | 6.2 |
| Injury of upper extremity nerves | 4301 | 5.2 |
| Complex soft tissue injury upper extremity | 27277 | 32.9 |
| Fracture pelvis | 15837 | 19.1 |
| Fracture hip | 104091 | 125.6 |
| Fracture femur shaft | 6908 | 8.3 |
| Fracture knee/lower leg | 35568 | 42.9 |
| Fracture ankle | 16146 | 19.5 |
| Fracture foot/toes | 19440 | 23.5 |
| Dislocation/sprain/strain knee | 17212 | 20.8 |
| Dislocation/sprain/strain ankle/foot | 8662 | 10.5 |
| Dislocation/sprain/strain hip | 2452 | 3.0 |
| Injury of lower extremity nerves | 490 | 0.6 |
| Complex soft tissue injury lower extremities | 5037 | 6.1 |
| Superficial injury, incl. contusions | 60979 | 73.6 |
| Open wounds | 61861 | 74.7 |
| Burns | 12475 | 15.1 |
| Poisoning | 52560 | 63.4 |
| Foreign body | 37506 | 45.3 |
| No injury after examination | 14468 | 17.5 |
| Other injury | 47405 | 57.2 |
